# The Structure of Metabolic Syndrome Components Across Follow-Up Survey From Childhood to Adolescence

**DOI:** 10.5812/ijem.4477

**Published:** 2012-12-21

**Authors:** Adeleh Bahar, Firoozeh Hosseini Esfahani, Mohammad Asghari Jafarabadi, Yadollah Mehrabi, Fereidoun Azizi

**Affiliations:** 1 Diabetes Research Centre, Mazandaran University of Medical Sciences, Sari, IR Iran; 2 Nutrition and Endocrine Research Center, Research Institute for Endocrine Sciences, Shahid Beheshti University of Medical Sciences, Tehran, IR Iran; 3 Medical Education Research Center, Department of Statistics and Epidemiology, Faculty of Health, Tabriz University of Medical Sciences, Tabriz, IR Iran; 4 School of Public Health, Shahid Beheshti University of Medical Sciences, Tehran, IR Iran; 5 Endocrine Research Center, Research Institute for Endocrine Sciences, Shahid Beheshti University of Medical Sciences, Tehran, IR Iran

**Keywords:** Adolescent, Childhood, Factor Analysis, Statistical

## Abstract

**Background:**

The choice of what parameters are needed for the diagnosis of Metabolic syndrome (MetS) has been criticized due to the lack of an actual “gold standard” diagnostic test even in adults. This problem seems to be greater in children and adolescents.

**Objectives:**

Stability assessment of factor structure underlying metabolic syndrome (MetS) components from childhood to adolescence in a panel study.

**Patients and Methods:**

A total number of 643 (305 boys and 338 girls) children (from 1999 to 2001), aged 6-10 years, with a complete median follow-up of 6.7 years (from 2006 to 2008) were selected among participants of Tehran Lipid and Glucose Study. We proposed 6 measured variables based on risk factors defined in Adult Treatment Panel III guidelines to describe clustering of MetS components.

**Results:**

The Goodness of fit of the two-factor model, extracted from exploratory factor analysis, was appropriate for boys and girls in both stages of the study using confirmatory factor analysis. Systolic blood pressure (SBP) and triglycerides (TGs), with parameter estimates (PE) of 1 and 0.75, respectively, were the greatest risk factors at baseline in boys and girls. Waist circumference with PE of 0.88 and 0.62, and SBP with PE of 0.99 and 0.86 in adolescent boys and girls, respectively, were important risk factors.

**Conclusions:**

Our panel study supports the stability of the two-factor six-variable model across two developmental stages from childhood to adolescence, among which adiposity, SBP, and TG were the predominant risk factors.

## 1. Background

Metabolic syndrome (MetS) is usually diagnosed using many different terms, owing in part to the lack of an actual “gold standard” diagnostic test even in adults (1). This problem seems to be greater in children and adolescents and the choice of what parameters are needed for the diagnosis of MetS has been criticized by several authors (2-4).

Exploratory factor analysis (EFA) has been applied to components of the MetS in several studies (5-7), identifying from one to seven distinct factors (5, 8-11). Most investigations performed so far have identified three or four factors, suggesting a possible heterogeneity of the MetS. Differences in results among various studies can be partly due to heterogeneity of the populations enrolled; some focused on very specific subgroups such as obese women (6) and some on adolescents (5, 8-11). Moreover, a major reason for discrepancies could be the difference in the list of parameters considered. Shah et al. concluded that the four-factor model of MetS including insulin resistance, obesity, lipids, and blood pressure was the most plausible model among the three competing models (12). In contrast to EFA, confirmatory factor analysis (CFA) is a theory-driven approach and can explicitly test whether or not the proposed constellation of components for a syndrome is best described by a single or more sets of underlying factors (9, 10, 13). Recently, most CFA studies have assessed the relationship of risk factors among adults or adolescents in a cross-sectional design (10, 14), and few studies (15) used CFA to test various hypothetical models in pre-adolescents and adolescents within a cohort study.

## 2. Objectives

Due to heterogeneity of the factor structures introduced in current literature, we assessed the stability of the factor structure underlying MetS components defined by the ATPIII (4) guidelines. In this panel study we used CFA to confirm the factor structure extracted by EFA and to test stability of these factors from childhood to adolescence.

## 3. Patients and Methods

### 3.1. Dataset and Subjects 

Tehran Lipid and Glucose Study (TLGS) is a prospective ongoing study to detect risk factors of non-communicable diseases among Tehran's urban population and to develop population-based measures and lifestyle modifications to decrease the prevalence and prevent the rising trends of diabetes mellitus and dyslipidemia (16, 17).

Initially in 1999-2001 a total number of 1165 boys and girls aged 6-10 years, under coverage of primary health care systems (the official bodies responsible for vaccination programs and collection of health-related statistics in a district), were selected using a multi-stage cluster random sampling method from municipality district No.13 of Tehran, the capital of Iran. The follow-up survey began in 2005 and was completed in 2008. After excluding subjects who lost to be followed-up, data of 643 individuals (305 boys and 338 girls) with a median follow-up of 6.7 years were used in this panel study. At the beginning of study and following approval of the ethics committee of Research Institute for Endocrine Sciences of Shahid Beheshti University of Medical Sciences, a written informed consent was obtained from parents of all participants. This study was conducted in accordance with principles of Declaration of Helsinki.

Using similar methods, data including fasting blood glucose (FBG), triglycerides (TGs), high density lipoprotein-cholesterol (HDL-C), blood pressure (BP), and anthropometric measurements were collected.

### 3.2. Measures of Risk Factors

Waist circumference (WC) was measured at umbilical level over light clothing using a non-stretchable measuring tape without any pressure to the body surface, being recorded to the nearest 0.1 cm. Participants were asked to remove tight or loose garments and belts intended to alter body shape; person performing the measurement inspected the tension of tape on the subject’s body to ensure that it had a proper tension (neither too loose nor too tight). To avoid subjective error, all measurements were taken by the same male physician for all males and the same female physician for all females.

Blood pressure was measured using a standard mercury manometer by certified technicians. The onset of the first (systolic) and fifth- phase (diastolic) Korotkoff sounds were recorded. Two measures were taken from each participant in a sitting position, and the average of readings was used for analysis.

To assay FBG and lipid levels of all participants, blood samples were collected between 7-9 A.M., after > 10-12 hours overnight fasting into evacuated tubes. Blood samples were drawn while the subjects were in a sitting position according to the standard protocol; the samples were centrifuged within 30–45 min after collection. All blood lipid analyses were performed at TLGS research laboratory on the day of blood collection. The analysis of samples was performed using a Selectra 2 auto-analyzer (Vital Scientific, Spankeren, Netherlands). Serum TG concentrations were assayed using commercially available enzymatic reagents (Pars Azmoon, Tehran, Iran) with glycerol phosphate oxidase. Samples were analyzed only when internal quality control assessments met the acceptable criteria. After precipitation of apolipoprotein B–containing lipoproteins with phosphotungstic acid, HDL-C was measured. Inter- and intra-assay coefficients of variation were 2 and 0.5% for HDL-C and 1.6 and 0.6% for TGs, respectively (18).

### 3.3. Definition of the MetS

As there is no universally accepted definition of metabolic syndrome, we used the definition as proposed by Cook et al. (19). This definition is based on criteria analogous to that of the National Cholesterol Education Program Expert Panel on Detection, Evaluation and Treatment of High Blood Cholesterol in Adult Treatment Panel III; it defines MetS as the presence of three or more of the following: fasting TG ≥ 110 mg/dL; HDL cholesterol ≤ 40 mg/dL; WC ≥ 90th percentile for age and sex, according to national reference curves (20); SBP and/or DBP >90th percentile for sex, age and height (21), from national reference cut-off points; and FBG ≥ 100 mg/dL.

### 3.4. Formulation of the Factor Structure of the MetS

Due to heterogeneity of the factor structures documented in current literature (18-22), we first explored factor structure by EFA and the best proposed structure entered in CFA. EFA was used to summarize variables by grouping inter-correlated variables; observed covariation between variables may be due to some underlying common factors. CFA evaluates whether the factors are correlated and also the magnitude of these correlations; therefore, the theory behind clustering of MetS risk factors structure was first explored in our population by EFA and then confirmed by CFA. In EFA we proposed 6 measured variables based on risk factors defined in the ATPIII guidelines (16) to describe clustering of MetS components; adiposity was defined by WC (22), lipid by HDL-C and TG levels (23), and BP factor by systolic (SBP) and diastolic BP (DBP) (13, 22).

### 3.5. Statistical Analysis

Before analysis, variables with high skewness or kurtosis (FBG and TG levels) were log transformed. Mean ± SD and proportions were presented for study subjects. Correlations between baseline anthropometric and metabolic variables were determined using Pearson correlation analysis. We performed EFA to explore the factor structure. The method of factor extraction was the principal component. The factors were rotated by varimax rotation. The number of factors to be retained was based on scree-plot analysis (factors above the break in the curve whose eigenvalues criteria were retained > 1). The resulting factor pattern was interpreted using factor loadings of ≥ 0.3, which cutoff value fulfills the minimum of the simplest structure possible; we only ignored 0.09 percent of information shared by factors and each variable. Proposed models were analyzed by means of CFA, based on the Bentler and Weeks theory (24). The maximum likelihood method was used to estimate parameter values and to test the significance at the 0.01 level. Goodness of Fit Chi-Square, Comparative Fit Index (CFI), and Standardized Root Mean Square (SRMR) were used for model fitting evaluation. Chi-Square test is often affected by the sample size and shows significant results for large samples; therefore two other indices (CFI and SRMR) were used as alternative fit evaluations (26). To examine components of risk factor variable of MetS, simultaneous multi-group analyses were conducted by factor loading in EFA; then for detection of the most risk factors in the MetS, we used CFA models. Data were analyzed by SPSS Inc, Chicago TL, Version 13, and CFA was performed using Statistica (Version 7; Statsoft.com).

## 4. Results

Of 643 subjects aged 6-10 years at baseline, 47.4% (n = 305) were boys. The mean ± SD and correlation coefficients of WC, SBP, DBP, HDL-C, and FBG are shown in Table 1. The highest correlation coefficient was observed for WC with SBP in adolescent boys (r = 0.53, P <0.01). The lowest correlation coefficient was seen between FBG and other variables, as was the factor loading of FBG (Table 2). The prevalence of individuals with MetS increased from 9.1 to 23.0% in boys and from 7.8 to 9.8% in girls. Results of EFA, assessed according to sex groups in both stages, are shown in Table 2. Six measured variables were reduced to two sets of inter-correlated factors, BP and adiposity/lipids, which together accounted for 56.8 and 58.4% of variance in measured variables among boys and 54.9 and 52% among girls, respectively in pre-adolescents and adolescents. HDL-C had a negative correlation with other metabolic risk factors with factor loadings ranging from -0.69 to -0.57 in boys and from -0.62 to 0.73 in girls in two developmental stages, respectively. All estimates of factor loadings were > 0.3, indicating an acceptable validity of measured variables.

**Table 1:** A Summary of Statistics and Correlations for Risk Factor Variables of Metabolic Syndrome by Sex in Children, Aged 6-10 Years at Baseline and at the Follow-Up Survey (6.7 Years Later): Tehran Lipid and Glucose Study

	Correlation Coefficient (r) (1999-2001), Children						Correlation Coefficient (r) (2006-2008), Adolescents						Prevalence of Some Parameters a, % (Mean ± SD)	
	WC	SBP	DBP	TGs	HDL-C	FBG	WC	SBP	DBP	TGs	HDL-C	FBG	1999-2001	2006-2008
** Boys (n=305)**														
WC a	1.00	0.28 b	0.06	0.49 b	-0.14 c	0.16	1.00	0.53 b	0.27 b	0.51 b	-0.29 b	0.03	5.7 (54.8 ± 6)	35.5 (78.9 ± 13)
SBP a		1.00	0.58 b	0.17 b	-0.07	0.12 c		1.00	0.54 b	0.32 b	-0.16 b	0.02	39.9 (105 ± 11)	17.2 (105 ± 11)
DBP a			1.00	0.11	-0.06	0.07			1.00	0.23 b	-0.17 b	-0.001	(71.7 ± 10)	(66.4±10)
TGs a				1.00	-0.35 b	0.11				1.00	-0.32 b	0.12 c	25.9 (93.3 ± 52)	35.1 (105.9±60)
HDL-C a					1.00	-0.14 c					1.00	0.06	29.0 (46.9 ± 11)	53.4 (41.7 ± 10)
FBG a						1.00						1.00	6.1 (86.7 ± 8)	6.0 (88.5 ± 7)
MetS a													9.1	23.0
**Girls (n=338)**														
WC a	1.00	0.19 b	0.15 b	0.33 b	-0.09	0.23 b	1.00	0.26 b	0.13^c^	0.35 b	-0.15 b	0.08	11.5 (56.5 ± 7)	15.5 (70.4 ± 9)
SBP a		1.00	0.64 b	0.12	0.15 b	0.14 c		1.00	0.47 b	0.13 c	-0.06	0.14 c	34.7 (102.5 ± 11)	10.4 (98.7 ± 11)
DBP a			1.00	0.07	0.08	0.04			1.00	0.13 c	-0.08	0.06	(70.6 ± 10)	(64.9 ± 9)
TGs a				1.00	-0.31 b	0.13 c				1.00	-0.26 b	0.08	25.2 (93.3 ± 52)	29.3 (101 ± 54)
HDL-C a					1.00	-0.03					1.00	0.05	28.9 (47.3 ± 11)	40.0 (44.0 ± 10)
FBG a						1.00						1.00	2.8 (84.8 ± 8)	3.0 (85.8 ± 7)
MetS a													7.8	9.8

^a^Prevalence of high WC, high BP, high TGs, low HDL-C, high FBG, and individuals with MetS, respectively.

^b^P < 0.01

^c^P < 0.05

Abbreviations: WC, Waist Circumference; SBP, Systolic Blood Pressure; DBP, Diastolic Blood Pressure; TGs, Triglycerides; HDL-C, High Density Lipoprotein-cholesterol; FBG, Fasting Blood Glucose; MetS, Metabolic Syndrome

**Table 2:** Factor Loadings for Risk Factor Variables of Metabolic Syndrome by Exploratory Factor Analysis in 643 Pre-adolescents (6-10 Years Old) at Baseline (1999-2001) and Adolescents at Follow-Up Survey (2006-2008): Tehran Lipid and Glucose Study

	Boys (n=305)				Girls (n=338)			
	1999-2001		2006-2008		1999-2001		2006-2008	
	Factor 1	Factor 2	Factor 1	Factor 2	Factor 1	Factor 2	Factor 1	Factor 2
**SBP **		0.87 a		0.78	0.89			0.82
**DBP **		0.84		0.77	0.85			0.78
**WC **	0.63		0.63			0.63	0.62	
**FBG **	0.44		0.53			0.42		0.37
**TGs **	0.80		0.82			0.77	0.80	
**HDL-C **	-0.69		-0.57			-0.62	-0.73	
**Variance, %**	28.9	27.9	29.7	28.7	28.8	26.1	26.4	25.6

^a^Factor loadings ≥ 0.3 are shown

**Figure 1:**
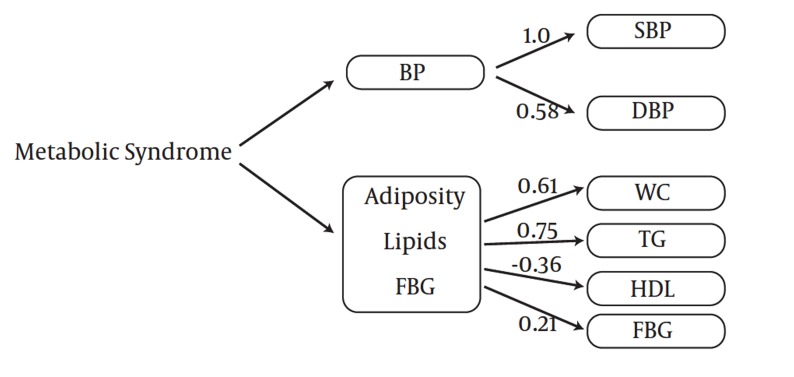
Metabolic Syndrome Factor Structure for Two Factors by Confirmatory Factor Analysis among 305 Pre-adolescent Boys at Baseline (1999-2001): Tehran Lipid and Glucose Study. X^2^=37.93; df =8.00, P=0.001; CFI=0.87; Standardized Root Mean Square Residual (SRMR) =0.07, P < 0.001 for all parameter estimates.Abbreviations: WC, Waist Circumference; BP, Blood Pressure; SBP, Systolic Blood Pressure; DBP, Diastolic Blood Pressure; TG, Triglycerides; FBG, Fasting Blood Glucose

**Figure 2:**
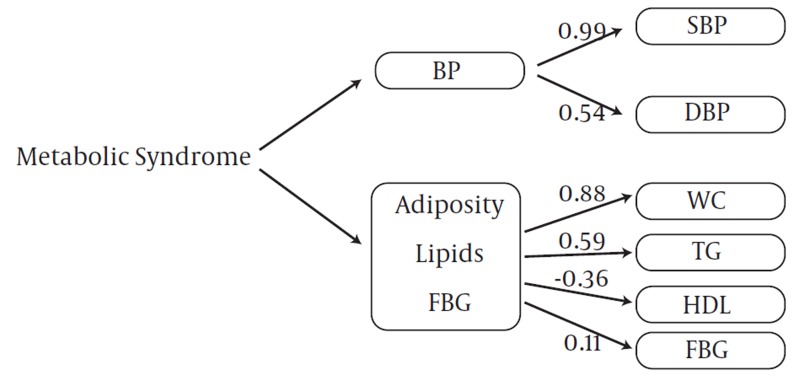
Metabolic Syndrome Factor Structure for Two Factors by Confirmatory Factor Analysis Models among 305 Adolescent Boys at the Follow-Up Survey (2006-2008): Tehran Lipid and Glucose Study. X2=23.83; df =7.00, P=0.001; CFI=0.95; Standardized Root Mean Square Residual (SRMR) =0.04, P < 0.001 for all parameter estimates. Abbreviations: WC, Waist Circumference; BP, Blood Pressure; SBP, Systolic Blood Pressure; DBP, Diastolic Blood Pressure; TG, Triglycerides; FBG, Fasting Blood Glucose

**Figure 3:**
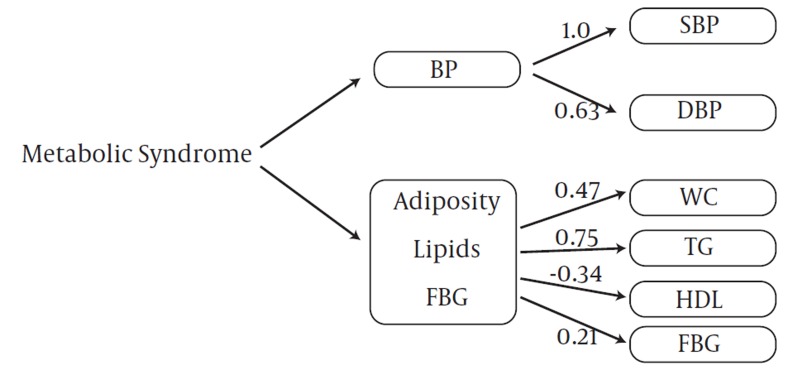
Metabolic Syndrome Factor Structure for Two Factors by Confirmatory Factor Analysis Models among 338 Pre-adolescent Girls at Baseline (1999-2001): Tehran Lipid and Glucose Study. X2=35.07; df=8.00, P=0.001; CFI=0.89; Standardized Root Mean Square Residual (SRMR) =0.073, P < 0.001 for all parameter estimates. Abbreviations: WC, Waist Circumference; BP, Blood Pressure; SBP, Systolic Blood Pressure; DBP, Diastolic Blood Pressure; TG, Triglycerides; FBG, Fasting Blood Glucose

**Figure 4:**
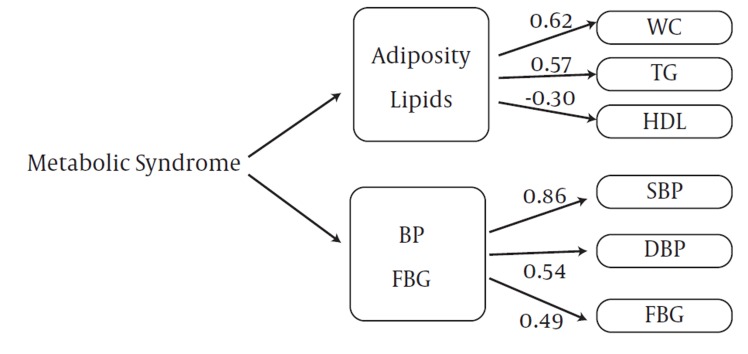
Metabolic Syndrome Factor Structure for Two Factors by Confirmatory Factor Analysis Models among 338 Adolescent Girls at the Follow-Up Survey (2006-2008): Tehran Lipid and Glucose Study. X^2^=12.99; df =7.00, P=0.07; CFI=0.96; Standardized Root Mean Square Residual=0.035, P < 0.001 for all parameter estimates except FBG. Abbreviations: WC, Waist Circumference; BP, Blood Pressure; SBP, Systolic Blood Pressure; DBP, Diastolic Blood Pressure; TG, Triglycerides; FBG, Fasting Blood Glucose

The goodness of fit of the two-factor model was appropriate for boys and girls in both stages. CFA with two-factor model analysis confirmed that SBP (PE =1) and TGs (PE = 0.75) were the greatest risk factors in boys at baseline (Figure 1), but these variables changed in the follow-up survey; SBP (PE = 0.99) and WC (PE = 0.88) were important metabolic risk factors with CFA in adolescent boys (Figure 2). FBG with PE ranging from 0.11–0.21 in adolescent and pre-adolescent boys had the lowest metabolic risk (Figure 1 and Figure 2). SBP and TGs, with PE = 1.0 and 0.75, respectively, ranked the first among metabolic risk factor structure for two-factor in CFA models among pre-adolescent girls at baseline (Figure 3); in the follow-up survey, SBP (PE = 0.86) and WC (PE = 0.62) were the most important risk factors, respectively (Figure 4).

## 5. Discussion

This panel study assessed the identification of components of MetS in Tehranian children and adolescents across two stages in TLGS using CFA in boys and girls separately, based on prior EFA. Results supported a two-factor structure in two stages in which SBP and TGs in a higher order associated with common factors representing MetS in boys and girls. In adolescents, adiposity had a higher association with MetS risk factors than TGs, and was more closely associated in boys than in girls.

There are few population-based studies that have evaluated the clustering of MetS risk factors among youths within a cohort study and across developmental stages (15). To our knowledge, this is the first investigation from the Middle-Eastern countries to report the clustering of pediatric MetS risk factors in a longitudinal design. Our study revealed only two factors: a BP factor and an adiposity/lipids factor; the study was in line with Bogalusa Heart study (25).

The BP loads onto a separate factor and is not related to other risk factors of MetS. The factor loadings and the percent of variance explained by BP were in line with Iranian population-based national study on children and adolescents (8), which showed that BP was a peripheral component far from the others, not among the core components, and was not related to other risk factors of MetS. Therefore, the percent of variance explained by BP is not due to explaining MetS; rather, it is due to BP factor which consists of SBP and DBP.

The constellation of central obesity, glucose intolerance, hypertension, and dyslipidemia, known as MetS, has been observed in a number of populations, worldwide (26-30). Adiposity is the predominant correlating risk factor of MetS (10, 15) and obesity epidemic has been the most important driving force for increasing the MetS (31). A previous study (27) showed that in adolescents, adiposity might be a stronger component of MetS than hyperinsulinemia. In our study, adiposity was a more sensitive factor in boys compared to girls and in adolescents compared to children.

In this study, TG levels had a stronger association with MetS risk factors than that of HDL-C among girls and boys in both stages. Elevated TG concentrations have been considered key markers for atherogenic dyslipidemia or lipid triad ( i.e. raised TG levels, small low density lipoprotein-cholesterol (LDL-C) particles, and low HDL-C (22)); low HDL-C was a component of MetS only in the presence of hypertriglyceridemia in patients with type 2 diabetes mellitus (10). Therefore, the use of TGs as a possible component of MetS in adolescents may be preferable measure of dyslipidemia.

There are weak associations between FBG and MetS risk factors in our study. The inverse factor loading of FBG on the first factor was shown in Goodman et al. (15) study and large representative Canadian study (25). In Fels longitudinal study, the proportion of change in FBG metric value was relatively stable from childhood to adolescence, when the level of this risk factor was in the normal risk category (32). The small factor loading of FBG in many of the models may attribute to a threshold effect in which FBG does not contribute to the pathologic clustering until it reaches to a certain threshold (15).

Goodmen et al. (15) assessed alternative hypothetical models for the factor structure of MetS across 3 developmental stages of the Fels longitudinal study. Among the models, measures of adiposity were most closely associated with MetS factors. In contrast to Fels longitudinal study, in which there was instability among models across pre-puberty, puberty, and post-puberty, in our study there was stability in wo-factor six-variable model across 2 developmental stages (childhood and adolescence). The model extracted from the previous EFA to ensure that highly correlated measures clustered together under separate factors, is consistent with the currently accepted definitions of MetS for pediatrics (33, 34), and supports its simplicity and applicability. MetS in IDF definition for children aged 10 years or older can be diagnosed by abdominal obesity (using waist circumference percentiles) and the presence of two or more of other clinical features (elevated TG, low HDL-c, high BP, increased FBG), and in WHO definition, MetS is present in patients with hyperinsulinemia or fasting blood glucose and at least two of the following: abdominal obesity, dyslipidemia or low HDL-C, and hypertension.

In a previous study by Li C et al. (10), based on CFA, there was a single underlying factor for four simple phenotypic traits, including WC, TGs, fasting insulin, and SBP, that may be plausible in adolescents. Fasting insulin and WC were almost equally associated with MetS suggesting that both insulin resistance and adiposity may be the key features of the syndrome. Our study examined MetS structure defined primarily by traditional risk factors except for fasting serum insulin. However, measuring insulin resistance is not routinely performed in clinical practice.

Some limitations in the present study may help point out directions for future research. Our study did not consider a number of non-traditional risk variables such as uric acid level, inflammation, pro-coagulation, and vitamin K dependent protein; based on recent researches, these may be indicative of MetS. Limitations of factor analysis that originate from several objective or arbitrary decisions should also be taken into account. Applying a modeling strategy with a longitudinal design from childhood and adolescence to adulthood may explore the complexity of interplaying MetS risk factors.

In conclusion, this panel study indicates that the two-factor six-variable structure underlying the clustering of MetS risk factors using CFA is stable across sex groups and two developmental stages from childhood to adolescence. SBP and TGs were associated with a highest common factor in children (boys and girls). In boys, adiposity was more closely associated with the clustering of MetS risk factors than in girls. Our findings support the current pediatric MetS definitions and can be applied in clinical practice and epidemiologic research as a simple and available measure to define MetS.

## References

[1] (2002). Third Report of the National Cholesterol Education Program (NCEP) Expert Panel on Detection, Evaluation, and Treatment of High Blood Cholesterol in Adults (Adult Treatment Panel III) final report.. Circulation..

[2] de Ferranti SD, Gauvreau K, Ludwig DS, Neufeld EJ, Newburger JW, Rifai N (2004). Prevalence of the metabolic syndrome in American adolescents: findings from the Third National Health and Nutrition Examination Survey.. Circulation..

[3] Ford ES, Li C (2008). Defining the metabolic syndrome in children and adolescents: will the real definition please stand up?. J Pediatr..

[4] Grundy SM, Cleeman JI, Daniels SR, Donato KA, Eckel RH, Franklin BA (2005). Diagnosis and management of the metabolic syndrome: an American Heart Association/National Heart, Lung, and Blood Institute Scientific Statement.. Circulation..

[5] Ghosh A (2007). Factor analysis of risk variables associated with metabolic syndrome in Asian Indian adolescents.. Am J Hum Biol..

[6] Lafortuna CL, Adorni F, Agosti F, Sartorio A (2008). Factor analysis of metabolic syndrome components in obese women.. Nutr Metab Cardiovasc Dis..

[7] Mannucci E, Monami M, Rotella CM (2007). How many components for the metabolic syndrome? Results of exploratory factor analysis in the FIBAR study.. Nutr Metab Cardiovasc Dis..

[8] Kelishadi R, Ardalan G, Adeli K, Motaghian M, Majdzadeh R, Mahmood-Arabi MS (2007). Factor analysis of cardiovascular risk clustering in pediatric metabolic syndrome: CASPIAN study.. Ann Nutr Metab..

[9] Lawlor DA, Ebrahim S, May M, Davey Smith G (2004). (Mis)use of Factor Analysis in the Study of Insulin Resistance Syndrome.. Am J Epidemiol..

[10] Li C, Ford ES (2007). Is there a single underlying factor for the metabolic syndrome in adolescents? A confirmatory factor analysis.. Diabetes Care..

[11] Wang JJ, Qiao Q, Miettinen ME, Lappalainen J, Hu G, Tuomilehto J (2004). The metabolic syndrome defined by factor analysis and incident type 2 diabetes in a chinese population with high postprandial glucose.. Diabetes Care..

[12] Shah S, Novak S, Stapleton LM (2006). Evaluation and comparison of models of metabolic syndrome using confirmatory factor analysis.. Eur J Epidemiol..

[13] Shen BJ, Todaro JF, Niaura R, McCaffery JM, Zhang J, Spiro A (2003). Are metabolic risk factors one unified syndrome? Modeling the structure of the metabolic syndrome X.. Am J Epidemiol..

[14] Pladevall M, Singal B, Williams LK, Brotons C, Guyer H, Sadurni J (2006). A single factor underlies the metabolic syndrome: a confirmatory factor analysis.. Diabetes Care..

[15] Goodman E, Li C, Tu YK, Ford E, Sun SS, Huang TT (2009). Stability of the factor structure of the metabolic syndrome across pubertal development: confirmatory factor analyses of three alternative models.. J Pediatr..

[16] Azizi F, Rahmani M, Emami H, Madjid M (2000). Tehran Lipid and Glucose Study.. CVD Prevention..

[17] Azizi F, Rahmani M, Emami H, Mirmiran P, Hajipour R, Madjid M (2002). Cardiovascular risk factors in an Iranian urban population: Tehran lipid and glucose study (phase 1).. Soz Praventivmed..

[18] Azizi F, Rahmani M, Madjid M, Allahverdian S, Ghanbili J, Ghanbarian A (2001). Serum lipid levels in an Iranian population of children and adolescents: Tehran lipid and glucose study.. Eur J Epidemiol..

[19] Kelishadi R, Cook SR, Motlagh ME, Gouya MM, Ardalan G, Motaghian M (2008). Metabolically obese normal weight and phenotypically obese metabolically normal youths: the CASPIAN Study.. J Am Diet Assoc..

[20] Kelishadi R, Gouya MM, Ardalan G, Hosseini M, Motaghian M, Delavari A (2007). First reference curves of waist and hip circumferences in an Asian population of youths: CASPIAN study.. J Trop Pediatr..

[21] (2004). The fourth report on the diagnosis, evaluation, and treatment of high blood pressure in children and adolescents.. Pediatrics..

[22] Leyva F, Godsland IF, Worthington M, Walton C, Stevenson JC (1998). Factors of the metabolic syndrome: baseline interrelationships in the first follow-up cohort of the HDDRISC Study (HDDRISC-1). Heart Disease and Diabetes Risk Indicators in a Screened Cohort.. Arterioscler Thromb Vasc Biol..

[23] Edwards KL, Austin MA, Newman B, Mayer E, Krauss RM, Selby JV (1994). Multivariate analysis of the insulin resistance syndrome in women.. Arterioscler Thromb..

[24] Bentler P, Weeks D (1980). Linear structural equations with latent variables.. Psychometrika..

[25] Chen W, Srinivasan SR, Elkasabany A, Berenson GS (1999). Cardiovascular risk factors clustering features of insulin resistance syndrome (Syndrome X) in a biracial (Black-White) population of children, adolescents, and young adults: the Bogalusa Heart Study.. Am J Epidemiol..

[26] Atabek ME, Pirgon O, Kurtoglu S (2006). Prevalence of metabolic syndrome in obese Turkish children and adolescents.. Diabetes Res Clin Pract..

[27] Misra A, Vikram NK, Arya S, Pandey RM, Dhingra V, Chatterjee A (2004). High prevalence of insulin resistance in postpubertal Asian Indian children is associated with adverse truncal body fat patterning, abdominal adiposity and excess body fat.. Int J Obes Relat Metab Disord..

[28] Morrison JA, Friedman LA, Harlan WR, Harlan LC, Barton BA, Schreiber GB (2005). Development of the metabolic syndrome in black and white adolescent girls: a longitudinal assessment.. Pediatrics..

[29] Scott LK (2006). Insulin resistance syndrome in children.. Pediatric nursing..

[30] Vikram NK, Misra A, Pandey RM, Luthra K, Wasir JS, Dhingra V (2006). Heterogeneous phenotypes of insulin resistance and its implications for defining metabolic syndrome in Asian Indian adolescents.. Atherosclerosis..

[31] Bays HE (2009). "Sick fat," metabolic disease, and atherosclerosis.. Am J Med..

[32] Li C, Ford ES, Huang TT, Sun SS, Goodman E (2009). Patterns of change in cardiometabolic risk factors associated with the metabolic syndrome among children and adolescents: the Fels Longitudinal Study.. J Pediatr..

[33] Alberti G (2006). The IDF consensus worldwide definition of the metabolic syndrome.. The IDF consensus worldwide definition of the metabolic syndrome..

[34] Balkau B, Charles MA, Drivsholm T, Borch-Johnsen K, Wareham N, Yudkin JS (2002). Frequency of the WHO metabolic syndrome in European cohorts, and an alternative definition of an insulin resistance syndrome.. Diabetes Metab..

